# Green Synthesis of Silver Nanoparticles with Culture Supernatant of a Bacterium *Pseudomonas rhodesiae* and Their Antibacterial Activity against Soft Rot Pathogen *Dickeya dadantii*

**DOI:** 10.3390/molecules24122303

**Published:** 2019-06-21

**Authors:** Afsana Hossain, Xianxian Hong, Ezzeldin Ibrahim, Bin Li, Guochang Sun, Youqing Meng, Yanli Wang, Qianli An

**Affiliations:** 1 State Key Laboratory of Rice Biology and Ministry of Agriculture Key Lab of Molecular Biology of Crop Pathogens and Insects, Institute of Biotechnology, Zhejiang University, Hangzhou 310058, China; afsana_07@yahoo.com (A.H.); 21816076@zju.edu.cn (X.H.); ezzelbehery8818@yahoo.com (E.I.); libin0571@zju.edu.cn (B.L.); 2 Department of Plant Pathology and Seed Science, Sylhet Agricultural University, Sylhet 3100, Bangladesh; 3 State Key Laboratory for Quality and Safety of Agro-products (in prepared), Institute of Plant Protection and Microbiology, Zhejiang Academy of Agricultural Sciences, Hangzhou 310021, China; sungc01@sina.com; 4 General Station of Plant Protection and Quarantine of Zhejiang Province, Hangzhou 310020, China; yqmeng_77@163.com

**Keywords:** green synthesis, silver nanoparticles, *Pseudomonas rhodesiae*, soft rot, sweet potato

## Abstract

Bacterial stem and root rot disease of sweet potato caused by *Dickeya dadantii* recently broke out in major sweet potato planting areas in China and calls for effective approaches to control the pathogen and disease. Here, we developed a simple method for green synthesis of silver nanoparticles (AgNPs) using bacterial culture supernatants. AgNPs synthesized with the cell-free culture supernatant of a bacterium *Pseudomonas rhodesiae* displayed the characteristic surface plasmon resonance peak at 420–430 nm and as nanocrystallites in diameters of 20–100 nm determined by transmission electron microscopy, scanning electron microscopy, and X-ray diffraction spectroscopy. Functional groups associated with proteins in the culture supernatant may reduce silver ions and stabilize AgNPs. The AgNPs showed antibacterial activities against *D. dadantii* growth, swimming motility, biofilm formation, and maceration of sweet potato tubers whereas the culture supernatant of *P. rhodesiae* did not. AgNPs (12 µg∙ml^−1^) and AgNO_3_ (50 µg∙ml^−1^) showed close antibacterial activities. The antibacterial activities increased with the increase of AgNP concentrations. The green-synthesized AgNPs can be used to control the soft rot disease by control of pathogen contamination of sweet potato seed tubers.

## 1. Introduction

Gram-negative bacteria belonging to the genera *Pectobacterium* and *Dickeya* are broad-host-range pathogens causing devastating soft rot diseases of ornamental and crop plants [1,2]. Some *Dickeya* species cause foot rot of rice [3,4], stalk rot of maize [5], blackleg and soft rot of potato [6,7,8], and stem and root rot of sweet potato [9,10], threatening staple food security.

Outbreaks of soft rot diseases of staple food crops in the last two decades worldwide indicate the failure in management of soft rot *Pectobacterium* and *Dickeya*. Crop varieties resistant to soft rot *Pectobacterium* and *Dickeya* are lacking while large-scale use of effective antibiotics is no longer allowed in fields due to the risks of introducing resistance to bacterial pathogens of humans or animals [2]. It is imperative to integrate conventional and innovative methods to effectively control the soft rot bacteria.

Nanoparticles with a size range of 1‒100 nm display unique properties of nanomaterials with a wide range of applications. Using nanoparticles as novel antimicrobial agents against broad-spectrum microbes including Gram-positive and Gram-negative multidrug-resistant bacteria, fungi, protozoa, and viruses has brought revolutions in the field of health, food, and agriculture technology. Physical, chemical, and biological methods have been developed to synthesize nanoparticles [11,12,13]. Nanoparticles produced by various methods have been recently tested against soft rot bacteria in vitro [14,15,16,17,18].

Green synthesis of nanoparticles, which uses eco-friendly and cost-effective reducing and stabilizing agents from plants, microbes, and other natural resources to produce nanoparticles without the use of toxic chemicals or stringent conditions, promotes the sustainable use of nanoparticles [12,13]. Green synthesis of nanoparticles with bacteria and cell-free extracts has shown advantages of easy handling, easy downstream processing, rapid scale-up processing, and easy genetic modification [19]. Extracellular polymeric substances produced by bacteria consist of polysaccharides, proteins, nucleic acids, uronic acids, and lipids. They contain functional groups, such as carboxyl, phosphoric, amine, and hydroxyl groups, which have adsorptive and adhesive properties and can serve as ligands and binding sites of metals [20,21]. Cell-free supernatants of bacterial cultures containing extracellular polymeric substances have been demonstrated to be effective for rapid, inexpensive, and safe syntheses of nanoparticles [22,23,24,25,26].

Outbreaks of bacterial stem and root rot disease of sweet potato caused by *D. dadantii* (former *Erwinia chrysanthemi*) [27] recently occurred in major sweet potato planting areas in China [9,10,28] and call for effective approaches to control the pathogen and disease. The aim of this study was to synthesize silver nanoparticles (AgNPs) using cell-free culture supernatants (CFCS) of plant growth-promoting bacteria (*Bacillus amyloliquefaciens* strain A3, *Paenibacillus polymyxa* strain ShX304, and *Pseudomonas rhodesiae* strain G1) and determine their antibacterial activities against the pathogen *D. dadantii*.

## 2. Results and Discussion

**Figure 1:**
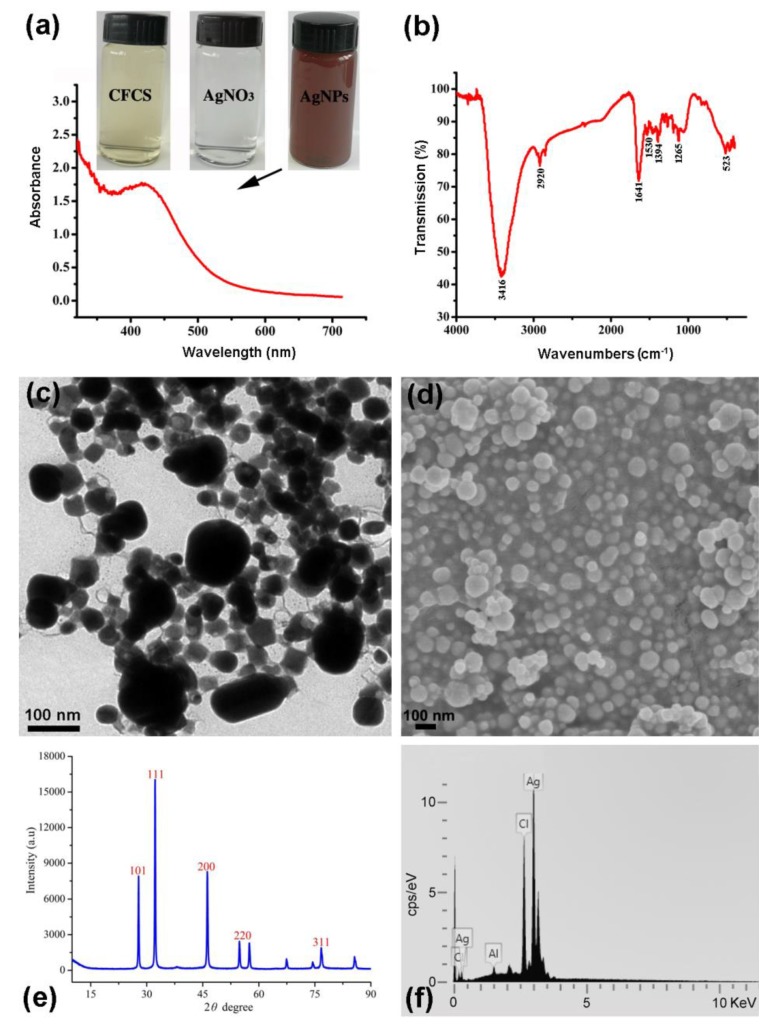
Characterization of silver nanoparticles (AgNPs) synthesized with cell-free culture filtrate (CFCS) of *Pseudomonas rhodesiae*. (**a**) UV–Visible absorption spectrum of the dark brown AgNP solution, which was formed by mixture of light yellow CFCS and colorless AgNO_3_ solution. AgNPs display a clear surface plasmon resonance peak at 420‒430 nm. (**b**) Fourier transform infrared spectrum showing functional groups responsible for the synthesis and stabilization of AgNPs. (**c**) Transmission electron micrograph showing AgNPs in spherical forms about 20‒100 nm in diameter. (**d**) Scanning electron micrograph showing AgNPs in spherical forms about 20‒100 nm in diameter. (**e**) X-ray diffraction spectrum showing the nanoscale size and crystalline nature of the AgNPs. (**f**) Energy dispersive spectrum showing the predominance of Ag element in the AgNP product.

AgNPs were synthesized by incubation of AgNO_3_ with CFCS of *P. rhodesiae* G1 and *B*. *amyloliquefaciens* A3 but not *Paenibacillus polymyxa* ShX304, indicated by the color change of the mixture from light yellow to dark brown (Figure 1a), as previous studies have shown [22,23,24,29]. The success and failure of production of AgNPs by incubation of AgNO_3_ with CFCS of different strains suggest that the components of CFCS determine the green synthesis of AgNPs.

CFCS [50% (*v/v*)] of *B*. *amyloliquefaciens* and *Paenibacillus polymyxa* generated clearing zones against *D*. *dadantii* in nutrient agar with diameters of 14.5 ± 0.3 mm and 12.0 ± 0.4 mm, respectively, including the diameter (7 mm) of the holes, whereas CFCS of *P. rhodesiae* did not. AgNO_3_ (50 µg∙mL^−1^), AgNPs (50 µg∙mL^−1^) synthesized with CFCS of *B*. *amyloliquefaciens*, and AgNPs (50 µg∙mL^−1^) synthesized with CFCS of *P. rhodesiae* generated clearing zones with diameters of 13.5 ± 0.4 mm, 17.0 ± 0.4 mm, and 22.0 ± 0.3 mm, respectively, including the diameter (7 mm) of the holes. AgNPs produced by incubation of CFCS of *Bacillus* bacteria including *B*. *amyloliquefaciens* [26,29] and their antibacterial activities have been demonstrated by previous studies [22,23,26,29,30,31]. Here, we showed that both CFCS of *B*. *amyloliquefaciens* and AgNPs synthesized with CFCS of *B*. *amyloliquefaciens* had antibacterial activities. On the other hand, CFCS of *P. rhodesiae* did not inhibit *D*. *dadantii* growth whereas AgNPs synthesized with CFCS of *P. rhodesiae* showed the strongest inhibition activity, suggesting that the antibacterial activity of AgNPs synthesized with CFCS of *P. rhodesiae* is independent of the components in CFCS binding to AgNPs. Therefore, to avoid interference with antibacterial activities by components of CFCS, we chose AgNPs synthesized with the CFCS of *P. rhodesiae* to characterize the green-synthesized AgNPs and determine their antibacterial activities against *D*. *dadantii*.

### 2.1. Characterization of AgNPs Synthesized with CFCS of P. Rhodesiae

AgNPs synthesized with CFCS of *P. rhodesiae* displayed a clear surface plasmon resonance peak at 420‒430 nm (Figure 1a) in the range of 350–450 nm for characteristic absorption peak of AgNPs by UV–Visible spectroscopy [32], as previous studies have shown [22,23,24,29].

The presence of functional groups in CFCS of *P. rhodesiae* responsible for the reduction of Ag^+^ and stabilization of the AgNPs was identified by FTIR spectroscopy. The FTIR spectrum shows absorption bands at wave numbers 3416, 2920, 1641, 1530, 1394, 1265, and 523 cm^−1^ (Figure 1b). The major band at 3416 cm^−1^ is attributed to N–H stretching vibrations; the distinctive peak at 1641 cm^−1^ is attributed to -C=O carbonyl group and –C=C stretching vibrations; the bands at 2920, 1530, 1394, and 1265 cm^−1^ are attributed to C–H stretching vibrations, C=N bond of Amide II, O–H deformation vibrations, and C–N stretching amine vibrations, respectively; the band at 523 cm^−1^ is attributed to C–Br stretching, which is characteristic of alkyl halides. The presence of these groups confirms the presence of proteins in CFCS of *P. rhodesiae* and suggests that these functional groups reduce Ag^+^ for the synthesis of AgNPs and bind to AgNPs for the stabilization of the nanoparticles [30,33,34,35].

The nanoscale size of the AgNPs synthesized with CFCS of *P. rhodesiae* was confirmed by transmission electron microscopy and scanning electron microscopy. The AgNPs were generally uniform and spherical about 20‒100 nm in diameter (Figure 1c,d).

The nanoscale size and crystalline nature of the AgNPs were confirmed by X-ray diffraction analysis. The X-ray diffraction spectrum of the product showed the characteristic Bragg reflection peaks of the Ag nanocrystallites at 2θ values of 32.23°, 46.19°, 54.78°, and 76.70°. The four peaks correspond to (1 1 1), (2 0 0), (2 2 0), and (3 1 1) (Figure 1e) crystalline planes of the face-centered cubic silver, which were identified by comparing with the standard powder diffraction card of Ag^0^ (JCPDS Card no. 04-0783) in the Joint Committee on Powder Diffraction Standards library [31,35]. The average particle size of the AgNPs (49.5 nm) can be calculated using Debye–Scherrer formula: *D* = *Kλ*/(*βCosθ*), where *D* is the average crystalline size of the nanoparticles, *K* is the Scherrer constant (0.94), *λ* is the X-ray wavelength (0.1546 nm), *β* is the full width at half maximum of the X-ray diffraction peak, and *θ* is the Bragg angle [33].

The predominance of Ag element in the AgNPs product was identified by energy dispersive spectroscopy. The AgNPs display typical optical absorption peak of Ag element at about 3 KeV (Figure 1f), as previous studies have shown [14,15,23,29,31,36]. The wt% of Ag, chlorine (Cl), and aluminium (Al) elements were about 83.1%, 16.1%, and 0.8%, respectively.

### 2.2. Antibacterial Activity of AgNPs against D. Dadantii

**Figure 2:**
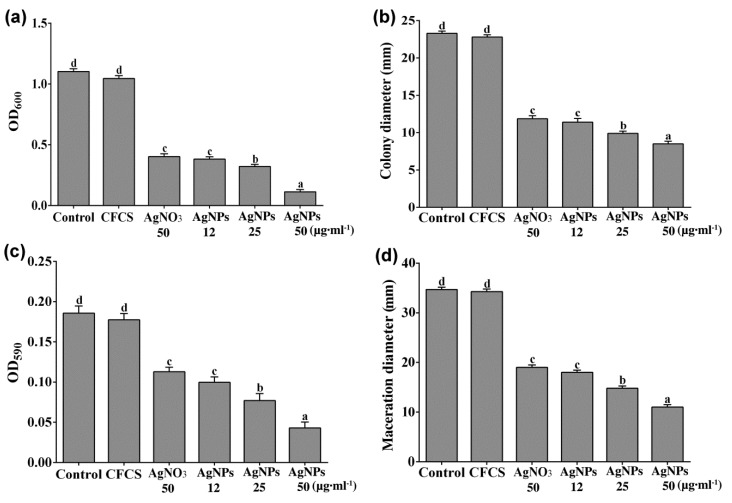
Antibacterial activity against *Dickeya dadantii* by silver nanoparticles (AgNPs) synthesized with cell-free culture filtrate (CFCS) of *Pseudomonas rhodesiae*. (**a**) *D. dadantii* growth in liquid nutrient broth containing CFCS (50%), AgNO_3_ (50 µg∙mL^−1^), or AgNPs (12, 25, or 50 µg∙mL^−1^) indicated by optical density at 600 nm (OD_600_). (**b**) Diameters of *D. dadantii* colonies formed on semisolid nutrient agar indicate *D. dadantii* swimming motility with CFCS, AgNO_3_, or AgNPs. (**c**) Crystal violet absorbance at 590 nm (OD_590_) indicates biofilms formed by *D. dadantii* with CFCS, AgNO3, or AgNPs. (**d**) Diameters of maceration tissues generated by *D. dadantii* in sweet potato tuber slices after immersing in CFCS, AgNO_3_, or AgNPs. Data are presented as mean values with standard errors (vertical bars); the different letters on the vertical bars indicate significant difference between treatments according to LSD test (*P* < 0.05).

**Figure 3:**
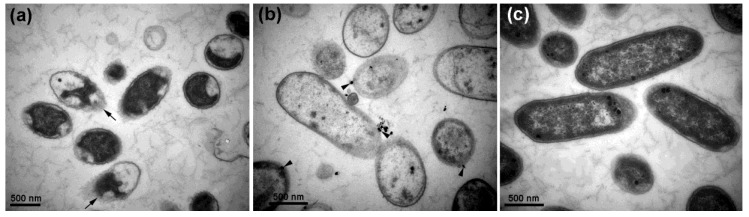
Transmission electron micrographs showing *Dickeya dadantii* cells treated by silver nanoparticles (50 µg∙mL^−1^) (**a**,**b**) and without treatment (**c**). (**a**) Coagulation and collapse of cytoplasm after treatment for 2 h. Arrows point to disintegration of cell envelopes. (**b**) Disintegration and clearing of cytoplasm after treatment for 6 h. Arrowheads point to silver nanoparticles adhering to cell surface. (**c**) Intact cell envelopes and cytoplasm filled in control cells without treatment.

CFCS of *P. rhodesiae* did not inhibit *D. dadantii* growth whereas AgNO_3_ and AgNPs significantly inhibited *D. dadantii* growth in the 24 h liquid culture (Figure 2a). Transmission electron microscopy revealed that after treatment with AgNPs (50 µg∙mL^−1^) for 2 h, most *D. dadantii* cells underwent cell death, indicated by coagulation and collapse of cytoplasm and disintegration of cell envelopes (Figure 3a). After treatment with AgNPs for 6 h, most *D. dadantii* cells were dead, indicated by disintegration and clearing of cytoplasm (Figure 3b). In contrast, most control cells had intact cell envelopes and cytoplasm filled in the cells (Figure 3c). Although most AgNPs were washed away with water and ethanol during preparation of bacterial cells for electron microscopy, a small number of AgNPs adhering to some bacterial cells were observed (Figure 3b).

*D. dadantii* grew and swam in the semisolid nutrient medium containing 0.3% agar and formed a halo about 23 mm in diameter after 48 h. CFCS of *P. rhodesiae* did not inhibit *D. dadantii* growth and swimming whereas AgNO_3_ (50 µg∙mL^−1^) and AgNPs significantly inhibited *D. dadantii* growth and swimming in the semisolid medium (Figure 2b).

*D. dadantii* cells formed biofilms on the surface of the polystyrene microplate wells during the 24 h incubation. CFCS of *P. rhodesiae* did not inhibit *D. dadantii* biofilm formation whereas AgNO_3_ and AgNPs significantly inhibited biofilm formation (Figure 2c).

*D. dadantii* degraded plant cell walls of the sweet potato tuber cells and generated maceration zones about 35 mm in diameter at 24 h after inoculation in the sweet potato slices. CFCS of *P. rhodesiae* did not inhibit tissue maceration by *D. dadantii* (Figure 2d) whereas AgNO_3_ and AgNPs significantly inhibited tissue maceration.

AgNO_3_ (50 µg∙mL^−1^) and AgNPs (12 µg∙mL^−1^) showed similar extents of inhibitions on *D. dadantii* growth, swimming motility, biofilm formation, and maceration of sweet potato tuber slices. The extents of inhibitions by AgNPs increased with the increase of AgNP concentrations (Figure 2). The in vivo inhibition of tissue maceration was consistent with the in vitro inhibition of bacterial growth, swimming motility, and biofilm formation.

AgNPs may serve as carriers to deliver Ag^+^ more effectively to bacteria membrane and cytoplasm [37]. The antibacterial activity of 20‒80 nm AgNPs was primarily assigned to the penetration of bacterial cell envelopes by Ag^+^ released from AgNPs [37,38,39,40]. The AgNPs synthesized with CFCS of *P. rhodesiae* are about 20‒100 nm in diameter and likely inhibit *D. dadantii* growth, swimming motility, and biofilm formation via adhering to bacterial cell surface, releasing toxic Ag^+^, and damaging cell membranes [41]. Ag^+^ penetrating into the *D. dadantii* cells may generate oxidative stress and bind to proteins and DNA to disturb respiration and DNA replication, leading to cell death [41,42].

*D. dadantii* movement and attachment to plant surfaces and formation of aggregates or biofilms on plant surfaces, in intercellular spaces, and in xylem vessels are important for survival and completing disease cycles [43,44]. AgNPs adhering to bacterial cell surfaces may inhibit *D. dadantii* movement and adhesion to plant surfaces; the following release of Ag^+^ may kill *D. dadantii* cells and remove biofilms.

Soft rot *Pectobacterium* and *Dickeya* bacteria are susceptible to AgNPs synthesized by different ways [14,15,16]. The latently infected seed tuber is one of the major sources of soft rot disease of sweet potato. Physical, chemical, and biological methods are explored to control the disease by avoiding and reducing tuber contamination to produce healthy crops. Here we show that AgNPs effectively inhibit *D. dadantii* growth and maceration of sweet potato tubers. Therefore, using AgNPs is a promising alternative for treatment of seed tubers to avoid and reduce tuber contamination.

In conclusion, we developed a simple method for green synthesis of AgNPs using bacterial culture supernatants. AgNPs synthesized with CFCS of *P. rhodesiae* show potent antibacterial activity against the pathogen *D. dadantii* and can be used to control *D. dadantii* contamination of seed tubers and produce healthy sweet potato crops.

## 3. Materials and Methods 

### 3.1. Bacteria

*D. dadantii* strain CZ1501, which causes bacterial stem and rot disease of sweet potato, was isolated from a stem of a sweet potato plant grown in Hangzhou, Zhejiang Province, China. *B. amyloliquefaciens* strain A3 isolated from rice seeds [45], *Paenibacillus polymyxa* strain ShX304 isolated from rhizosphere soils of cotton plants [46], and *P. rhodesiae* strain G1 isolated from garlic plants were cultured for green synthesis of AgNPs.

### 3.2. Synthesis of AgNPs

Strains A3, Sx3, or G1 were cultured in nutrient broth (10 g tryptone, 3 g beef extract, 2.5 g glucose, and 5 g NaCl per liter; pH 7.0) at 30 °C and 200 rpm for 24 h. CFCS was obtained by centrifugation and confirmed by no bacterial growth after incubating 100 µL of supernatants on nutrient agar (15 g agar per liter) at 30 °C for 24 h. CFCS (35 mL) and freshly prepared aqueous AgNO_3_ (Cat. no. 10018461; Sinopharm, Shanghai, China) solution (1.0 mM) (115 mL) were mixed in a 250 mL Erlenmeyer flask, kept in the dark, and shaken at 200 rpm and 30 °C for 48 h. Nutrient broth (35 mL) and AgNO_3_ solution (1.0 mM) (115 mL) were mixed and used as control. Synthesis of AgNPs was monitored by color change from light yellow to dark brown. The dark brown solution (2 mL) was mixed with Milli-Q water (2 mL) and analyzed by UV–Visible spectrometry from 200 to 800 nm at 1 nm resolution using a Shimadzu UV-2550 spectrometer (Shimadzu, Kyoto, Japan) [24,29]. The dark brown solution containing AgNPs was centrifuged at 27,200× *g* for 10 min and the pellet was washed twice with Milli-Q water and freeze-dried. Freeze-dried AgNPs were weighed and a 50 mg∙mL^−1^ stock solution dissolved in Milli-Q water was prepared.

### 3.3. Characterization of AgNPs Synthesized with CFCS of P. Rhodesiae

Functional groups responsible for the synthesis and stabilization of AgNPs were detected by Fourier transform infrared (FTIR) spectroscopy. Freeze-dried AgNP powders (1 mg) were mixed with KBr (300 mg) and compressed to thin pellets by hydraulic pellet press. FTIR spectra were recorded in the range of 500‒4000 cm^−1^ with a resolution of 4 cm^−1^ using an AVATAR 370 FTIR spectrometer (Thermo Nicolet, MA, USA).

The size and morphology of AgNPs were observed by scanning electron microscopy and transmission electron microscopy. One drop of the AgNP solution was applied onto a carbon-coated copper grid and dried under a lamp. AgNPs carried by the grid were observed with an SU8010 field emission scanning electron microscope (Hitachi, Tokyo, Japan) and a JEM-1230 transmission electron microscope (JEOL, Tokyo, Japan). The silver element of AgNPs was detected by an X-Max N energy dispersive spectrometer (Oxford Instruments, Oxford, UK) at 20 keV.

The crystalline nature of AgNPs was analyzed by X-ray diffraction spectroscopy. Freeze-dried AgNP powders were applied onto a coated film on a glass slide and analyzed using a D8 Advance Diffractometer (Bruker, Karlsruhe, Germany) in the 2θ range from 20° to 80° with Cu-Kα radiation at 40 kV and 40 mA.

### 3.4. Antibacterial Assays

*D. dadantii* CZ1501 grown to midexponential phase was suspended with nutrient broth to about 5 × 10^8^ CFU∙mL^−1^ before use.

Antibacterial activities against *D. dadantii* were first determined by the diffusion assay with agar plates. *D. dadantii* suspension was inoculated into the nutrient agar at about 45 °C to 10^7^ cells∙mL^−1^; holes (7 mm in diameter) were made in the agar plates with sterilized steel punchers; CFCS (50%) from cultures of the three plant growth-promoting strains, aqueous AgNO_3_ solution (50 µg∙mL^−1^), and aqueous AgNP solution (50 µg∙mL^−1^) were loaded into the holes and incubated for 24 h [47]. Antibacterial activities were evaluated by the diameters of the clearing zones formed around the holes. This experiment was done with three replications and repeated three times.

Antibacterial activities against *D. dadantii* growth in nutrient broth were further determined. *D. dadantii* suspension (100 µL) was inoculated into nutrient broth (5 mL) and used as control. Equal volumes of CFCS of *P. rhodesiae* and nutrient broth were mixed; *D. dadantii* suspension (100 µL) was added into the mixture (5 mL). AgNO_3_ was added to nutrient broth to the final concentration of 50 µg∙ml^−1^. AgNPs synthesized with CFCS of *P. rhodesiae* were added into nutrient broth to final concentrations of 12, 25, and 50 µg∙mL^−1^. *D. dadantii* suspension (100 µL) was inoculated into the nutrient broth (5 mL) containing AgNO_3_ or AgNPs. The cultures were shaken at 200 rpm and 30 °C for 24 h and optical density at 600 nm (OD_600_) was measured using a SpectraMax spectrophotometer (Molecular Devices, Sunnyvale, CA, USA). This experiment was done with three replications and repeated three times.

The swimming motility of *D. dadantii* was determined with semisolid nutrient agar [0.3% (*w/v*)]. Equal volumes of CFCS of *P. rhodesiae* and a semisolid nutrient agar [0.6% (*w/v*)] were mixed at about 45 °C. AgNO_3_ was added into a semisolid nutrient agar [0.3% (*w/v*)] to the final concentration of 50 µg∙mL^−1^. AgNPs synthesized with CFCS of *P. rhodesiae* were added to final concentrations of 12, 25, and 50 µg∙mL^−1^. *D. dadantii* suspension (5 µL) was spotted onto the center of the semisolid nutrient agar in Petri dishes and incubated at 30 °C for 48 h. The diameters of the colonies formed by *D. dadantii* were measured. This experiment was done with three replications and repeated three times.

Biofilm formed by *D. dadantii* was stained by crystal violet and quantified by absorbance at 590 nm (OD_590_). *D. dadantii* suspension (100 µL) was mixed with nutrient broth (100 µL) and used as control. AgNO_3_ was added into nutrient broth to the concentration of 100 µg∙mL^−1^. AgNPs synthesized with CFCS of *P. rhodesiae* were added to concentrations of 24, 50, and 100 µg∙mL^−1^. *D. dadantii* suspension (100 µL) was mixed with CFCS of *P. rhodesiae* (100 µL) or the nutrient broth (100 µL) containing AgNO_3_ (100 µg∙mL^−1^) or AgNPs (24, 50, or 100 µg∙mL^−1^). The mixtures were separately added into wells of a 96-well microplate while the nutrient broth was used as blank. After incubating at 30 °C for 24 h, the liquid in the wells was removed. The wells were washed three times with distilled water and then air-dried for 1 h. The bacterial cells attached to the wells were stained with 1 % (*w/v*) crystal violet (200 µL) for 30 min at room temperature. The wells were then washed thoroughly with distilled water and air-dried for 1 h. The crystal violet dye that stained the bacterial cells in the wells was dissolved with 33% (*v/v*) acetic acid (200 µL) and OD_590_ was measured. This experiment was done with six replications and repeated three times.

In vivo antibacterial activity against *D. dadantii* was determined with sweet potato tuber slices. Sweet potato tubers were surface-sterilized with 70% (*v/v*) ethanol, washed with sterile distilled water, and cut into slices (10 mm in thickness). The tuber slices were immersed in distilled water (control), 50% (*v/v*) CFCS, AgNO_3_ solution (50 µg∙ml^−1^), or AgNP solutions (12, 25, or 50 µg∙mL^−1^) for 1 h, and then put into Petri dishes and air-dried for 1 h. Afterwards, *D. dadantii* suspension (5 µL) was spotted in a puncture at the center of the tuber slice and incubated at 30 °C for 24 h. Diameters of the maceration zones around the punctures were measured. This experiment was done with six replications and repeated three times.

### 3.5. Transmission Electron Microscopy

The effect of AgNPs on *D. dadantii* cell structure was determined by transmission electron microscopy. *D. dadantii* suspension and *D. dadantii* suspension containing AgNPs (50 µg∙mL^−1^) synthesized with CFCS of *P. rhodesiae* were incubated at 30 °C with shaking at 200 rpm for 2 h and 6 h. *D. dadantii* cells were harvested by centrifugation and washed twice with 0.1 M phosphate buffer (pH 7) and then fixed in 2.5% (*v/v*) glutaraldehyde in the phosphate buffer at 4 °C overnight. After washing with the phosphate buffer, *D. dadantii* cells were fixed in 1% (*w/v*) OsO_4_ dissolved in the phosphate buffer for 1 h at room temperature. *D. dadantii* cells were then washed with distilled water and dehydrated by a graded series (50%, 70%, 80%, 90%, 95%, and 100%) of ethanol. Dehydrated *D. dadantii* cells were infiltrated by Spurr’s resin at room temperature and embedded in Spurr’s resin at 70 °C for 9 h. Ultrathin sections were cut with glass knives on an ultramicrotome (Reichert-Jung, Vienna, Austria), collected with copper grids, stained with uranyl acetate and lead citrate, and observed with the JEM-1230 transmission electron microscope.

### 3.6. Statistical Analysis

Statistical analyses were done using the SPSS software version 16 (SPSS, Chicago, IL, USA) and the significance was set at *P* < 0.05.
